# Phonon transport in Janus monolayer siblings: a comparison of 1T and 2H-ISbTe

**DOI:** 10.1039/d2ra08100h

**Published:** 2023-02-01

**Authors:** Viet-Ha Chu, Tien-Ha Le, Truong-Tho Pham, Duc-Long Nguyen

**Affiliations:** a Department of Physics, TNU-University of Education Thai Nguyen 250000 Vietnam; b Institute of Sciences and Technology, TNU-University of Sciences Thai Nguyen Vietnam; c Laboratory of Applied Physics, Science and Technology Advanced Institute, Van Lang University Ho Chi Minh City Vietnam nguyenduclong@vlu.edu.vn; d Faculty of Applied Technology, School of Technology, Van Lang University Ho Chi Minh City Vietnam

## Abstract

In the last decade, two-dimension materials with reduced symmetry have attracted a lot of attention due to the emerging quantum features induced by their structural asymmetry. Two-dimensional Janus materials, named after the Roman deity of beginnings and endings who has two faces, have a structure with broken mirror symmetry because the two sides of the material have distinct chemical compositions. Extensive study has been undertaken on phonon transport for Janus monolayers for their strong applicability in thermoelectrics compared to their parent material, while Janus materials with the same space group but a distinct crystal protype have received very little attention. Using first-principles calculations and the Boltzmann transport equation accelerated by a machine learning interatomic potential, we explore the phonon transport of 1T and 2H-ISbTe. ISbTe possesses significant intrinsic phonon–phonon interactions, resulting in a low lattice thermal conductivity, as a result of its covalent bonding and low elastic constants. A thorough examination of phonon group velocity, phonon lifetime, and heat carrier identification reveals that 2H has a low lattice thermal conductivity of 1.5 W mK^−1^, which is 2.3 times lower than its 1T sibling. This study demonstrates Janus ISbTe monolayers have extensive physical phenomena in their thermal transport characteristics, which might provide a new degree of control over their thermal conductivity for applications such as thermal management and thermoelectric devices.

## Abstract 2

The phonon transport characteristics of Janus ISbTe siblings are investigated using first-principles calculations and the Boltzmann transport equation accelerated by a machine learning interatomic potential.

## Graphical abstract

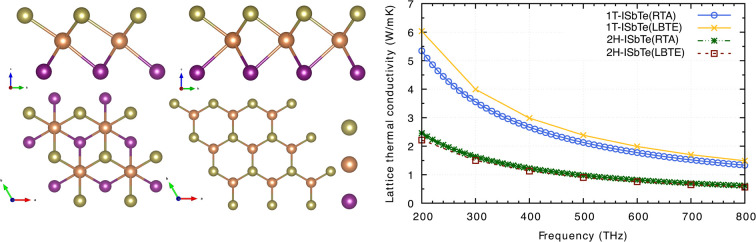

## 1 Introduction

By allowing the transformation of waste heat into electricity, thermoelectric (TE) materials are viewed as playing a crucial role in meeting the current worldwide push for green and sustainable energy.^1–4^ There have been multiple discoveries of extremely efficient bulk TE materials thanks to the decade of continued study into thermoelectrics.^5–11^ Theoretically, increasing the TE figure of merit (*ZT*) by alloying, doping, or nanostructuring is the most effective way to improve the performance of TE materials.^12^ By definition, *ZT* = *S*^2^*σT*/*κ*, where *S* is the Seebeck coefficient, *σ* is the electrical conductivity, *κ* is the overall thermal conductivity (a sum of electronic (*κ*_e_) and lattice (*κ*_L_) contributions), and *T* is the absolute temperature. The potential of low-dimensional materials has attracted a lot of attention in this field because of the significance of nanostructuring in hindering lattice heat transfer in bulk materials. Low lattice thermal conductivities at room temperature have been reported for a number of two-dimensional (2D) materials, including stanene,^13^ phosphorene,^14^ MoS_2_, MoSe_2_,^15^ and WSe_2_.^16^

When it comes to the potential applications of the aforementioned 2D materials, transition metal dichalcogenides (2D-TMDs) stand out because of their distinctive structure and semiconducting characteristics. These less complex compounds, including SnS_2_, SnSe_2_ and SnSe,^17,18^ combine chemical stability, low toxicity, and earth abundance to potentially produce high thermoelectric performance 2D materials. Janus monolayers, in which the chalcogen atoms in each layer are bonded to atoms of a different element, are a more sophisticated type of 2D-TMD structure that are the subject of extensive research in this field.^19,20^ Because of the existence of two distinct chalcogen surfaces, Janus monolayers of TMDs are so named after the Roman god of transitions as they are unable to sustain out-of-plane mirror symmetry. When compared to their classical counterparts, Janus monolayers have a number of benefits. These include the lack of inversion and mirror symmetry, the presence of a vertical enormous polarization field, and a whole new set of characteristics. Electronics, optoelectronics, and piezoelectronics are only a few of the areas where Janus monolayers have been proposed for use. Regarding thermal transport, it has been predicted that the lattice thermal conductivities of Janus MoSSe/SnSSe/PtSSe 2D-TMDs will be much lower than those of the MoS_2_/SnS_2_/PtS_2_ monolayers.^21–23^ These results are consistent with the common knowledge that a more complicated unit cell results in greater phonon scattering and a lower thermal conductivity. Specifically, Guo *et al.* estimated the thermal conductivity of MoSSe monolayers by solving the phonon Boltzmann equation in the single-mode relaxation time approximation, and found that it is intermediate between that of MoS_2_ and MoSe_2_ monolayers.^21^ The lattice thermal conductivity of the Janus SnSSe monolayer at room temperature is ≈1.5 times lower than SnS_2_, according to first-principles calculations by Liu *et al.*^22^ Patel *et al.* found that Janus WSSe and WSTe monolayers had much lower lattice thermal conductivities than their parent compound WS_2_ monolayers.^24^ The thermal transport of 2D Janus materials with the same symmetry but a different crystal prototype has received surprisingly little attention from researchers. There is significant scientific interest in elucidating the thermal characteristics of Janus monolayers since solving these issues might have far-reaching implications for the use of Janus 2D materials in the future. We investigate the thermal conductivity of the lattice in two Janus monolayers of ISbTe that share the same space group *P*3*m*1, 2H-ISbTe and 1T-ISbTe. Due to its lower group velocity and shorter phonon lifetime, we found that 2H-ISbTe has a lattice thermal conductivity that is just half that of its sibling.

The remaining parts of the article are structured as described below. In the following section, the computational details of the electronic structures, and electron and phonon transports are presented. In the third part, we will discuss the ISbTe monolayer's elastic characteristics, electronic structures, and phonon transports. In the final part of this article, the fourth segment, we will provide our conclusions.

## 2 Computational methods

The electronic structures of ISbTe monolayers were performed using density functional theory.^25,26^ The exchange and correlation interactions are treated using the Perdew–Burke–Ernzerhof (PBE) form of the generalized gradient approximation^27^ as implemented in Quantum ESPRESSO.^28–30^ The kinetic energy cutoffs for the wave functions and charge density are 40 Ry and 320 Ry, respectively. We utilised a 15 Å vacuum layer in the *z* direction so that there would be no contact between the monolayers when we applied periodic boundary conditions. Atoms and lattices were relaxed according to the Hellman–Feynman forces using the conjugate gradient method during structural optimization. This process continued until the atomic forces were reduced to a value that was lower than 0.001 eV Å^−1^. The first Brillouin zone (BZ) was sampled with 13 × 13 × 1 Monkhorst–Pack *k*-point grid.^31^ The Phonopy code^32^ is utilised in the calculation of phonon dispersion relations as well as harmonic force constants. Through the use of the MLIP software,^33^ moment tensor potentials, often known as MTPs, are trained to assess phononic properties. Simulations of *ab initio* molecular dynamics (AIMD)^34^ are run with a time step of 1 ps for 5 × 5 × 1 supercells with *k*-point mesh 3 × 3 × 1. The preparation of the training sets necessary for the formation of MTPs is accomplished by running two independent AIMD simulations, one from 0 to 200 K and the other from 200 to 700 K for 1000 time steps each. Following each individual calculation, a subsample of 1800 trajectories was selected for inclusion in the whole training set while the other 200 subsamples are used for the test set. We then used the trained MTPs to construct anharmonic third-order interatomic force constants by calculating for 5 × 5 × 1 supercells without imposing any cutoff pair-distance (which required force calculations for 1899 structures with 75 atoms for both cases 2H and 1T-ISbTe). Direct solution of linearized phonon Boltzmann equation^35^ together with the single mode relaxation time approximation (RTA) with *q*-point sampling meshes 101 × 101 × 1 are used to calculate the lattice thermal conductivity with the Phono3py package.^36^ In order to keep the rotational symmetry of free space intact, the second-order IFCs are corrected using the Hiphive package,^37^ which ensures that the out-of-plane (ZA) branch is strictly quadratic around the *Γ* point. The Born–Huang^38^ sets of linear constraints, commonly known as sum rules, are used by the Hiphive implementation to impose rotational symmetry on the Cartesian representation of IFCs. It should be kept in mind that the *z*-coordinate of the unit cell length affects the computed lattice thermal conductivities for a 2D material. The calculated result needs to be multiplied by the factor *L*_d_/*d*, where *L*_d_ is the length of the unit cell along the *z*-axis and *d* is the sum of the 2D material's thickness, *h*, and the van der Waals radii of the outermost atoms of the monolayer.

## 3 Main results and discussion

**Fig. 1:**
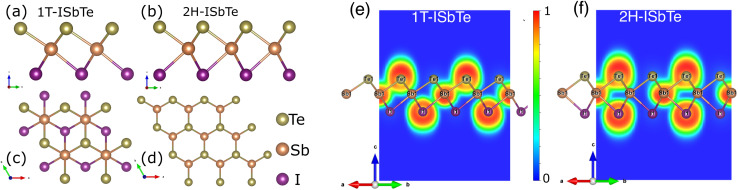
(a, b) Top and (c, d) side view of the crystal structures of 1T and 2H-ISbTe. The yellow balls represent Te atoms, and the orange and violet balls are Sb and I atoms respectively. (e) and (f) are the calculated electron localization functions of 1T and 2H-ISbTe along the plane parallel to (110) and cutting through I–Sb–Te atoms. The color bar represents the value of the ELF from 0 to 1.

The Janus monolayer ISbTe, unlike ordinary 2H-TMD materials, has no reflection symmetry with regard to the centre Sb atoms since the outermost atom has been replaced. the crystal structures of the 1T and 2H-ISbTe monolayers are shown in Fig. 1. 1T-ISbTe has a trigonal omega-derived structured and crystallizes in the trigonal *P*3*m*1 space group. The monolayer consists of one ISbTe sheet oriented in the *z* direction. Sb is bonded to three equivalent Te and three equivalent I atoms to form edge-sharing SbTe_3_I_3_ octahedra. All *d*_Sb–Te_ bond lengths are 3.01 Å. All *d*_Sb–I_ bond lengths are 3.22 Å. Te atoms are bonded in a distorted T-shaped geometry to three equivalent Sb atoms. Iodine atoms are bonded in a distorted T-shaped geometry to three equivalent Sb atoms. The calculated lattice constant of 1T-ISbTe is 4.32 Å, with the shown orientation, Te atoms located on top, Sb atoms at the centre, and I atoms at the bottom forming a single layer thickness of 3.71 Å. A summary of the basic crystal structures of the 2 phases of ISbTe is shown in Table 1. On the other hand the 2H-ISbTe has a similar 2H-TMD crystal structure, it is a molybdenite-derived structured and crystallizes in the trigonal *P*3*m*1 space group. Sb atoms are bonded to three equivalent Te and three equivalent I atoms to form distorted edge-sharing SbTe_3_I_3_ pentagonal pyramids. All *d*_Sb–I_ bond lengths are 3.28 Å. Te atoms are bonded in a distorted T-shaped geometry to three equivalent Sb atoms. I atoms are bonded in a 3-coordinate geometry to three equivalent Sb atoms, as summarized in Table 1.

**Table 1:** Lattice constants *a* (Å), the Sb–Te and Sb–I bond lengths *d* (Å), thickness *h* (Å), and the energy band gap *E*_gap_ (eV) of ISbTe 2H and 1T monolayers

ISbTe	*a* (Å)	*d*_Sb–Te_(Å)	*d*_Sb–I_(Å)	*h*(Å)	*E*_gap_ (eV)
1T	4.322	3.01	3.22	3.710	1.15
2H	4.192	3.02	3.28	4.018	1.21

To further examine the bonding features of Sb–I and Sb–Te, the electron localization function (ELF) for ISbTe monolayers has been determined. The ELF typically indicates the following: the negative position, depicted in blue, is the electron divergence region, where electrons are lost, and the positive position, depicted in yellow, is the electron convergence region, where electrons are gained.^39^ The covalent bond electrons converge toward the centre of the two atoms, whereas the ionic bond electrons are biassed toward one side. Fig. 1(e and f) depict the ELF along the (110) plane, where ELF values range from 0 to 1. The results indicate that the ELF values of both case 1T and 2H-ISbTe exhibit similar characteristics. At the atomic sites, the electron localization force (ELF) is high, while in the bonding regions it is lower, the electron localises between Sb and Te with an ELF value of 0.695, whereas the value of the region between Sb and I atoms is only 0.43. In the case of covalent bonds, it is known that the bonding electrons are delocalized, whereas lone-pair electrons are concentrated near to the atoms. Sb–Te and Sb–I are thus bonded by covalent bonds, with Sb–I bonds being weaker. Strong covalent bonds can result in significant intrinsic phonon–phonon scattering, which in turn can result in low lattice thermal conductivities in ISbTe monolayers.

To determine whether or not the ISbTe monolayers are able to maintain their mechanical integrity, we calculate their elastic constants by fitting the strain-energy relationship as described in ref. 40. Because of the hexagonal symmetry, there are 2 independent elastic constants denoted by *C*_11_ and *C*_12_ and *C*_66_ = (*C*_11_ − *C*_12_)/2 to form the stiffness matrix:
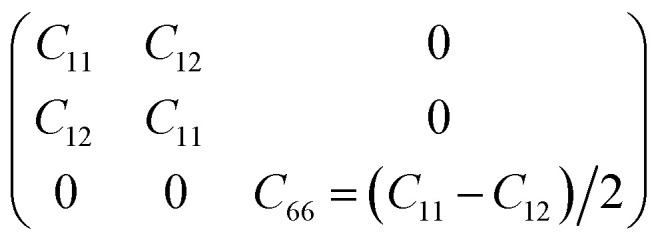


Table 2 shows the elastic constants *C*_*ij*_, Young's modulus *Y*^2D^, shear modulus *G*^2D^ in units of (N m^−1^) and the dimensionless Poisson's ratio *ν* of 1T and 2H-ISbTe. The 2D Young's moduli (in-plane stiffness) for strains in the Cartesian [10] and [01] directions are (here *C*_11_ = *C*_22_)^41^1
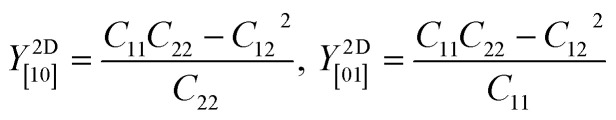


**Table 2:** Calculated elastic constants *C*_*ij*_, Young's modulus *Y*^2D^, shear modulus *G*^2D^ in (N m^−1^) and the dimensionless Poisson's ratio *ν* of 1T and 2H-ISbTe

ISbTe	*C*_11_ = *C*_22_	*C*_12_	*C*_66_ = *G*^2D^	*Y* ^2D^_[10]_ = *Y*^2D^_[01]_	*ν*_[10]_ = *ν*_[01]_
1T	27.038	7.791	9.624	24.793	0.288
2H	29.090	15.265	6.913	21.080	0.525

The corresponding dimensionless Poisson's ratios are2
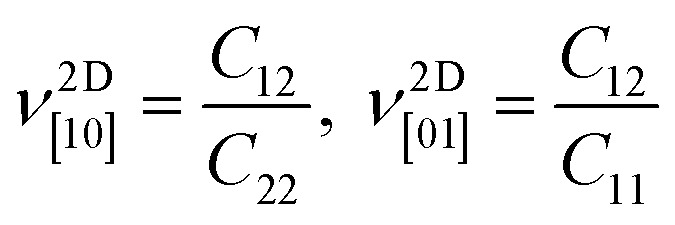
and the 2D shear modulus is

3 *G*^2D^ = *C*_66_

We found *C*_11_ = 27.038 N m^−1^, *C*_12_ = 7.791 N m^−1^ and *C*_66_ = 9.624 N m^−1^ for 1T-ISbTe which are in accordance with the Born requirements for the mechanical stability of the material:^41^*C*_11_ > 0, *C*_11_ > |*C*_12_| and *C*_66_ > 0. The values are 29.090 N m^−1^, 15.265 N m^−1^ and 6.913 N m^−1^ for 2H-ISbTe as summarized in Table 2. The result are in good agreement with literature results^42^ which demonstrates the accuracy of our present calculations. The Young's modulus’ for 1T and 2H-ISBTe are 24.793 and 21.080 N m^−1^ respectively which are smaller than that in Janus MoXY (X/Y = S, Se, and Te) (102–134) N m^−1^ (ref. 21 and 43) and comparable to BiXY (X = S, Se, Te and Y = F, Cl, Br, I).^44^ As a result of their low Young's modulus’, ISbTe monolayers may readily undergo significant in-plane strain engineering, which is crucial for fine-tuning the material's physical characteristics through strain. The Poisson's ratios *ν* of Janus ISbTe monolayers are isotropic, the calculated values are 0.288 and 0.525 for 1T and 2H-ISbTe respectively.

**Fig. 2:**
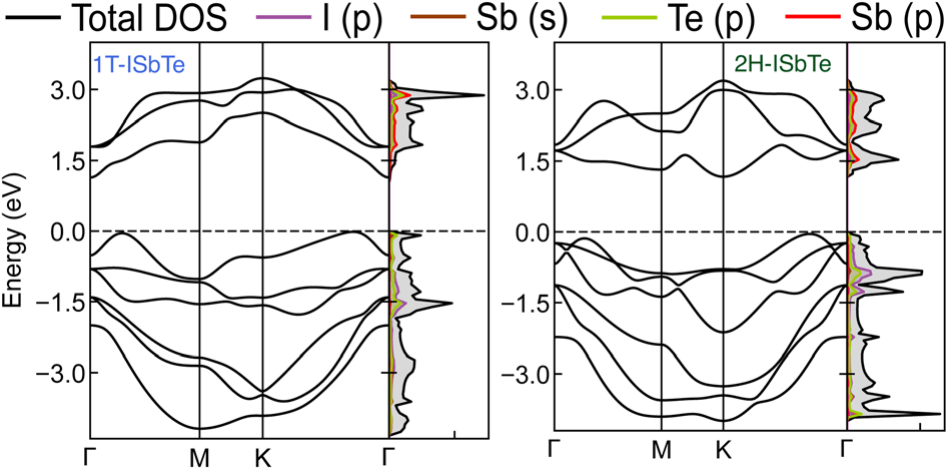
Electronic band structures and projected density of states of Janus 1T (left) and 2H-ISbTe (right). The Fermi level is set to the valence band maximum (VBM).

The electronic band structures of Janus 1T and 2H-ISbTe are shown in Fig. 2. As we can clearly see, these sibling Janus monolayers are indirect band gap semiconductors with the valence band maximum (VBM) located in the middle of K–*Γ*. While 1T-ISbTe has the conduction band minimum (CBM) located at the *Γ* point to form an indirect band gap of 1.15 eV (at the PBE accuracy level), in contrast 2H-ISbTe has its CBM located at the K point to form an indirect band of 1.21 eV, which is somewhat higher than that of the 1T structure and in good agreement with previous theoretical studies.^45,46^ There is an additional band valley between *Γ* and *M*, which is visible in both cases and may contribute to better thermoelectric performance.^47^ While 2H-ISbTe has a somewhat lower band valley, the 1T-ISbTe valley is almost degenerate with the VBM. The valley in 1T-ISbTe enables it to have a more pronounced density of states (DOS) peak around the Fermi level than 2H-ISbTe, where the p-orbital is prominent.

**Fig. 3:**
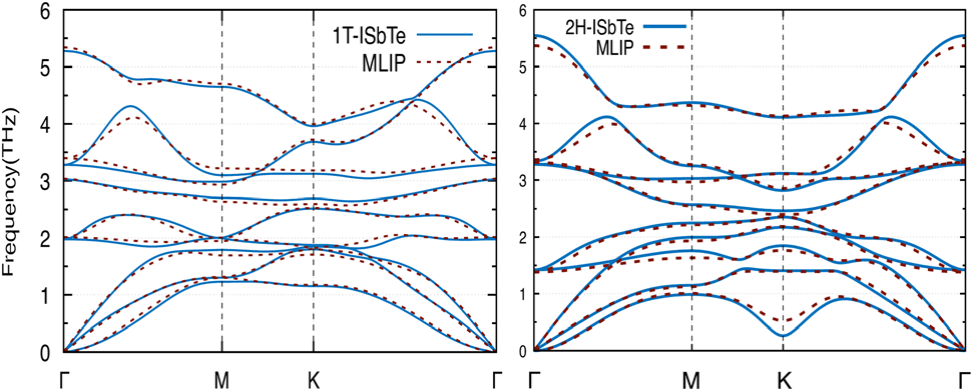
Phonon dispersion curves of 1T-ISbTe (left) and 2H-ISbTe (right) monolayers. The solid lines and dash lines represent the DFT and MLIP results respectively.

We now discuss the lattice dynamics of these two Janus systems. At first glance, the phonon dispersion of 1T-ISbTe shows no imaginary frequency modes indicating its dynamical stability as shown in Fig. 3. The computed phonon dispersion curves of the trained machine-learning interatomic potentials are also displayed on the same figure to demonstrate the trained model's precision. With the exception of a few unavoidable differences, the two curves almost coincide, illustrating the precision of the trained MLIP. Having 3 atoms in a primitive cell, the phonon dispersion of 1T-ISbTe consists of 3 acoustic and 6 optical phonon branches. The acoustic branch in the *z*-axis (ZA) is quadratic, which is typical of 2D materials,^48–50^ whereas the LA and TA branches are linear in the vicinity of the *Γ* point.

The maximum phonon mode frequency of 1T-ISbTe is 5.26 THz, which is somewhat lower than that of 2H-ISbTe. Acoustic phonon modes of 1T-ISbTe span up to 1.85 THz and are nearly entangled with the optical modes at the *K* point, whereas the acoustic modes of 2H-ISbTe span to the same values and are substantially separated from the optical modes. At the *K* point, the ZA mode of 2H-ISbTe exhibits a softening characteristic. The first optical modes of 1T-ISbTe at *Γ* degenerate at 1.98 THz, which is higher than those of 2H-ISbTe at 1.42 THz. These variations will be discussed in view of their potential to provide insight on the lattice thermal conductivity behaviour in these two systems. In both systems, the lattice thermal conductivity may benefit from the wide dispersion of the two highest optical modes.

Lattice thermal conductivity can be obtained as follows^36^4
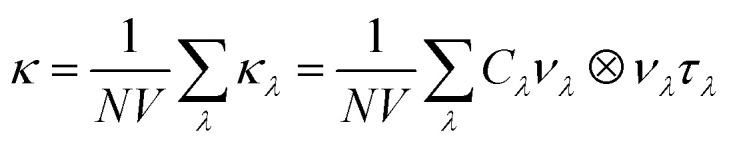
where *λ* is the phonon mode, *N* is the total number of *q* points sampling BZ, *V* is the volume of a unit cell, and *C*_*λ*_, *ν*_*λ*_, *τ*_*λ*_ are the specific heat, phonon velocity, and phonon lifetime, respectively. The phonon lifetime *τ*_*λ*_ can be calculated by the phonon linewidth 2*Γ*_*λ*_(*ω*_*λ*_) of the phonon mode *λ*:5
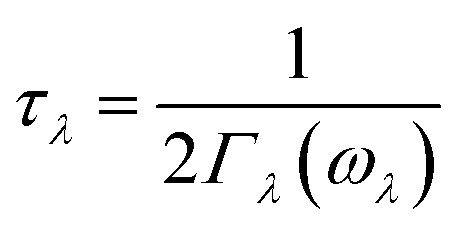


in which *Γ*_*λ*_(*ω*) takes the form analogous to the Fermi golden rule:6
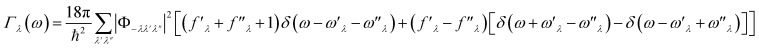
where *f*_*λ*_ is the phonon equilibrium occupancy and *Φ*_−*λλ*′*λ*′′_ denotes the strength of interaction among the three phonons *λ*, *λ*′, and *λ*′′ involved in the scattering.

**Fig. 4:**
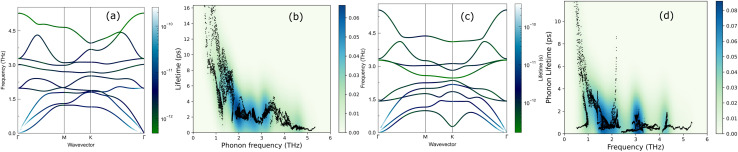
Phonon dispersion decorated with the phonon lifetime for 1T (a) and 2H-ISbTe (c), and phonon lifetime distribution as a function of frequency of the two phases 1T and 2H (b and d). The color bar represents the density of data points.

**Fig. 5:**
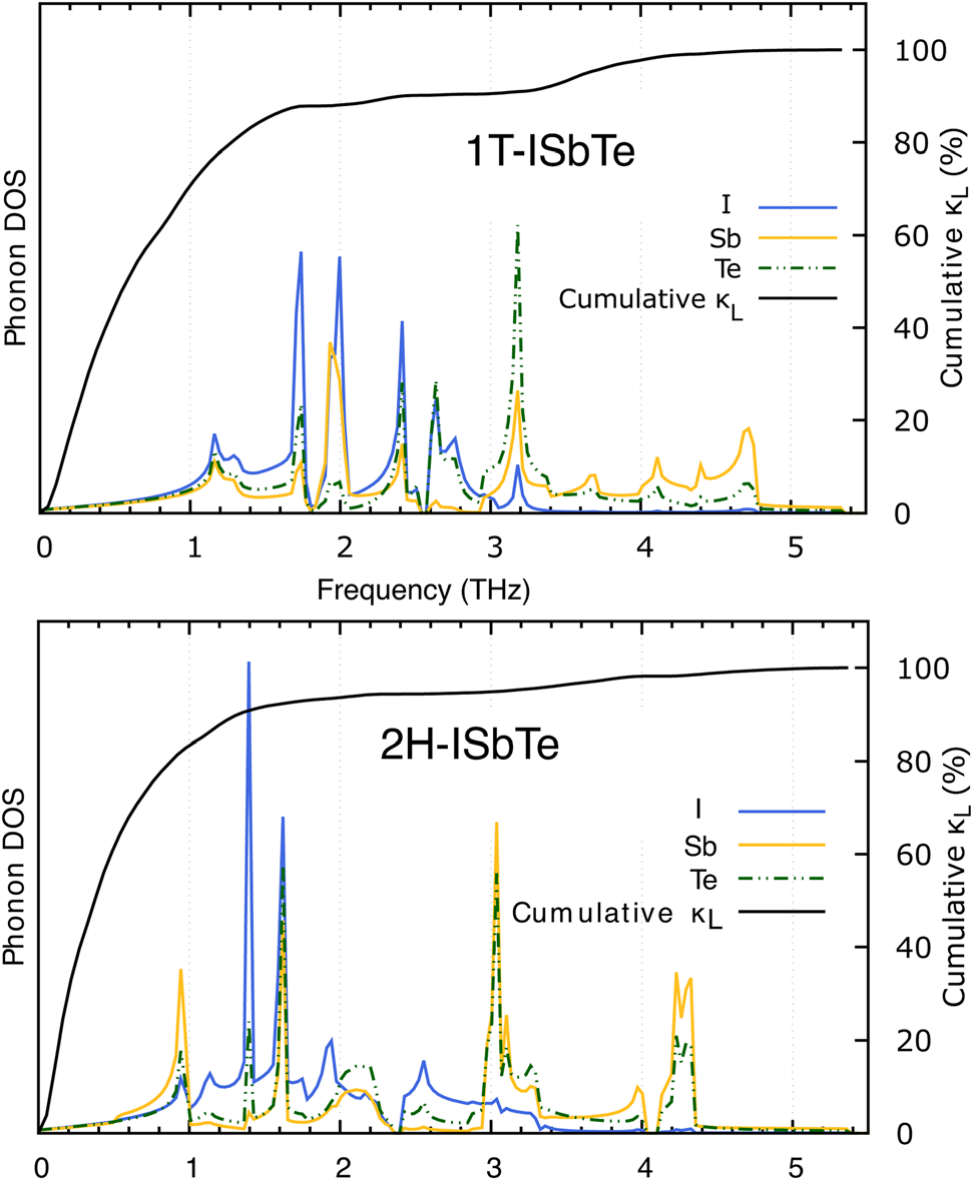
Cumulative *κ*_L_ of 1T-ISbTe (top) and 2H-ISbTe (bottom) as a function of phonon frequency.

The lifetimes of phonons are directly related to the *κ*_L_. Therefore, the calculated phonon lifetime as a function of frequencies is shown in Fig. 4. The phonon modes are shown as black dots on a coloured background, with regions of high phonon mode density indicated in blue and low density in white. In 1T-ISbTe, from 0 THz onwards, the phonon lifetime rapidly decreases with frequency, with an intermediate increase and a drop between 1 THz and 1.7 THz. In the range of 1.7–3.4 THz, the average phonon lifetime is around 2.5 picoseconds. We can see from the phonon lifetime distribution that 1T-ISbTe has very low phonon lifetimes, the maximum phonon lifetime of 1T-ISbTe at 300 K is determined to be 276.8, 240.4, and 112.5 ps for ZA, LA, and TA modes, respectively, whereas the mean values are 16.7, 13.5, and 7.7 ps for the aforementioned modes. 75% of the phonon lifetime of the ZA, LA, and TA modes of 1T-ISbTe is less than 9.1, 12.3, and 7.86 ps, respectively. The mean value of phonon lifetime for the optical modes is only 2.08 ps. In 1T-ISbTe, the *κ*_L_ is lowered because the phonons have short lifetimes, leading to increased phonon–phonon scattering rates. The longest phonon lifetimes are seen between 0.8 and 1.7 THz. In accordance with the phonon DOS presented in Fig. 5, the phonon lifetimes are concentrated in the 0.8–1.7 THz, 1.7–3.4 THz, and 4.4–4.8 THz ranges. Since there are several phonon modes concentrated at these epoch-defining points (blue or white-green backgrounds), phonon annihilation is the end outcome of the massive phonon–phonon scattering or Umklapp processes that take place there. Three acoustic modes mostly contribute to the total *κ*_L_, with the LA phonon mode contributing the most at 33.5%, followed by TA and ZA at 29.9% and 24.4%, respectively. It is important to note that the 8^th^ optical mode contributes up to 7.6% of the *κ*_L_, which is uncommon compared to other thermoelectric materials in which optical modes contribute rarely. This observation is consistent with Fig. 5, which depicts the contribution of the vibration modes of each atom to the *κ*_L_ of the two phases 1T-ISbTe. The behaviour of the large dispersion of high phonon frequencies likewise reflects this completely. In contrast to its sibling 1T, 2H-ISbTe exhibits a distinctive phonon lifetime distribution due to its distinct phonon dispersion property. The LA mode has a maximum phonon lifetime of 597 ps, but the ZA and TA modes have substantially lower lifetimes of 174.7 and 152 ps, respectively. The maximum values appear to be higher than those of the 1T structure, however the mean values of the three acoustic modes are only 8.9, 8.3, and 2.84 ps, which is considerably less than the 1T case. For ZA, LA, and TA modes, 75% of the phonon lifetime is determined to be less than 5.5, 4.8, and 2.0, respectively. The average phonon lifetime of the optical modes of 2H-ISbTe is only 0.79 ps, which is much lower than that of the 1T case, highlighting the distinction between the two structures. This numerical analysis is visibly supported in Fig. 4(b and d) for the two cases, which depicts the substantially lower concentration of phonon lifetime in the 2H case compared to the 1T scenario.

**Fig. 6:**
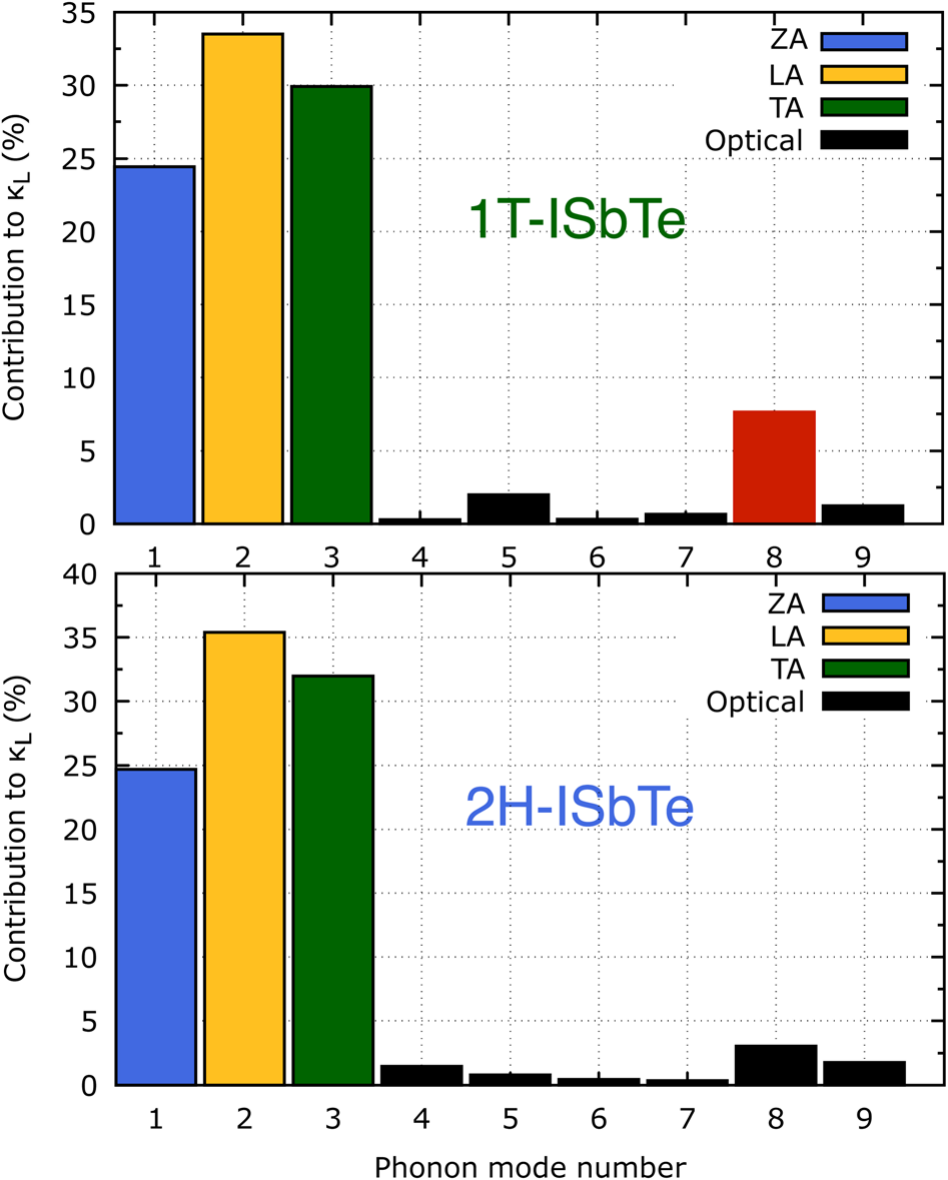
Phonon mode contribution to the total lattice thermal conductivity of 1T (top) and 2H-ISbTe (bottom). The 8^th^ optical mode's contribution of 1T-ISbTe is shown in red color.

**Fig. 7:**
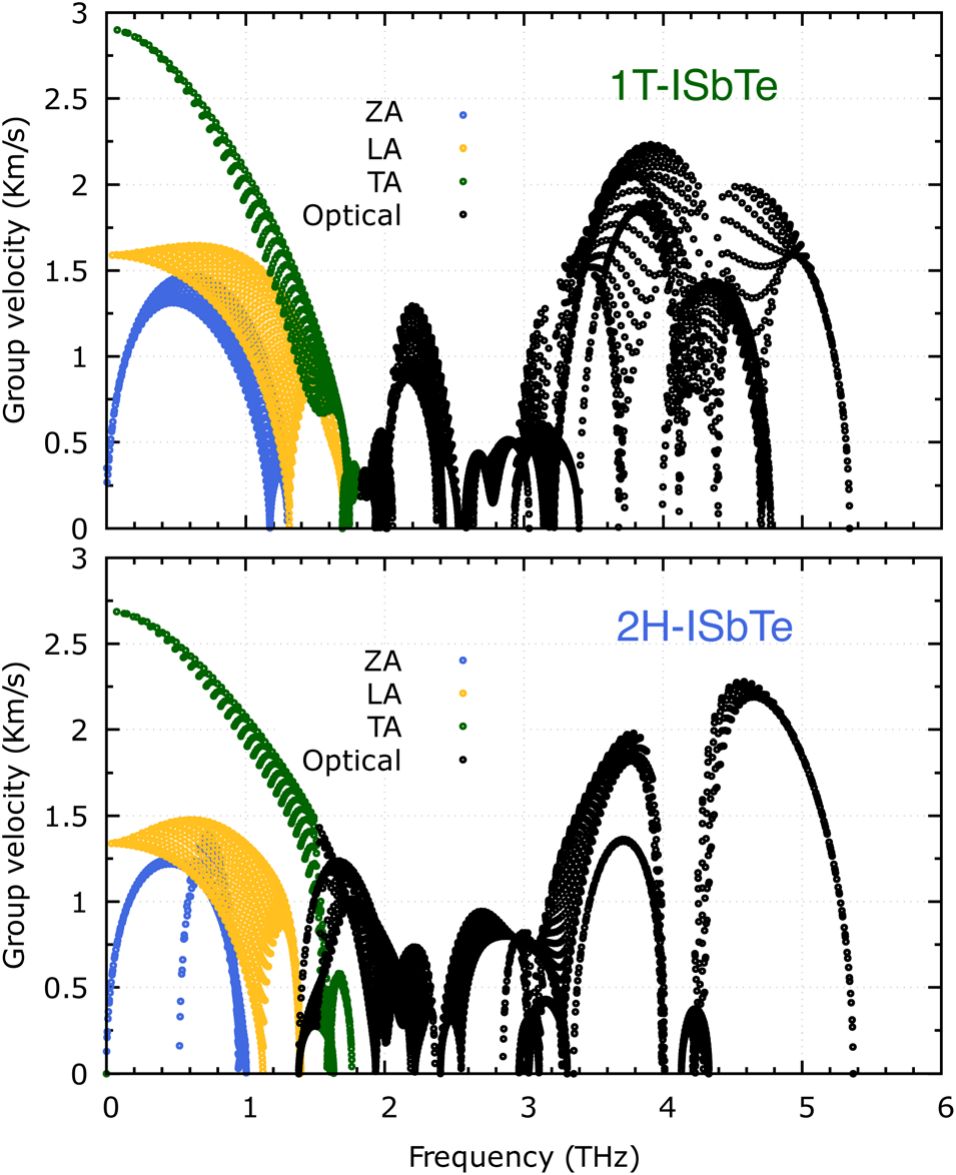
Calculated phonon group velocity of 1T (top) and 2H-ISbTe (bottom). The group velocity of ZA, LA, TA and optical modes are represented by blue, yellow, green, and black hollow circles, respectively.

It is also fascinating to examine how phonon lifetime varies across the Brillouin zone across the various phonon branches. As can be seen in Fig. 4(a and c), the phonon lifetime of the three acoustic modes is longest around the *Γ* point and decreases monotonically as one moves closer to the *M* and *K* zone boundaries. Despite sharing certain essential features with 1T-ISbTe in terms of phonon transport, the 2H structure's *κ*_L_ is likely to be substantially lower due to a number of key differences. We start with the similar characteristics, that is the contribution to the total *κ*_L_ coming mostly from the acoustic modes, as shown in Fig. 6 where the LA mode contributes 35.4%, followed by the TA mode and ZA mode with 32% and 24.7%, respectively. It also has a similar distribution of phonon lifetime across the Brillouin zone, where the large values can be found near the zone center and shortening when coming near to the zone boundaries, as shown in Fig. 4. However, the 2H-ISbTe has lower group velocities of the three most important modes, the acoustic ones, with the maximum group velocities of the ZA, LA, and TA modes being 1.39, 1.48 and 2.69 km s^−1^ as compared to 1.47, 1.65 and 2.90 km s^−1^ for 1T-ISbTe, respectively (see Fig. 7). Unique compared to its brother, the softening phonon mode ZA near *K* exhibits an additional waterfall group velocity at around 0.5 THz; this behaviour significantly reduces the overall group velocity in 2H-ISbTe. In both structures, the optical modes exhibit fascinating behaviour, in which their group velocity is equivalent to or greater than that of their acoustic mode, which is rare among 2D materials.^51–53^ The maximum group velocity for the optical modes of 1T-ISbTe is determined to be 2.23 km s^−1^, which is even greater than its LA and ZA modes, resulting in a contribution of up to 7.63% to the *κ*_L_ from the 8^th^ optical mode in this structure.

To further explore the heat carrier identity on Janus ISbTe monolayers, we studied the detailed vibration modes and their correlation to the *κ*_L_. Cumulative lattice thermal conductivity with respect to frequency is defined as:7
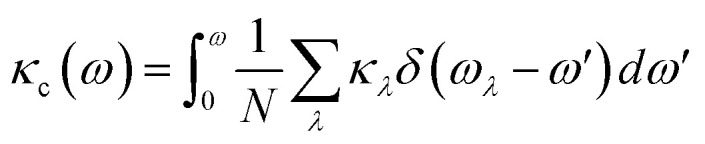
The thermal conductivity of the two monolayers 2H and 1T-ISbTe together with phonon density of states (PDOS) are shown in Fig. 5. The first peak in the PDOS spectrum of 1T-ISbTe is located at 1.2 THz, with contributions from I, Sb, and Te atoms, with iodine atoms contributing the most. This low-frequency range contributes up to about 80% of the total *κ*_L_, indicating the importance of low-frequency collective vibrations of atoms to the heat transfer in this material, especially the iodine vibrations. The mid-frequency region (1.2–3.1 THz) possesses five notable peaks of I atoms vibrations and two prominent peaks of Sb atom vibrations, but Te atoms display less noticeable peaks. Although this mid-frequency comprises the majority of 1T-ISbTe's vibrations, it contributes relatively little to *κ*_L_. Te and Sb atom vibrations with frequency dominated the region above 3 THz, whereas I atoms displayed weaker peaks. This region adds the remaining 20% to the total lattice thermal conductivity of 1T-ISbTe. In contrast, the vibration of 2H-ISbTe reveals a different scenario. The initial peak of the low frequency range (below 1 THz) is composed of three components, although Sb vibrations contribute the most, and this region accounts for more than 80% of the total *κ*_L_. Other peaks in the PDOS spectrum have little impact on *κ*_L_. This suggests that the majority of heat carriers in 2H-ISbTe are Sb atoms, as opposed to 1T-ISbTe.

**Fig. 8:**
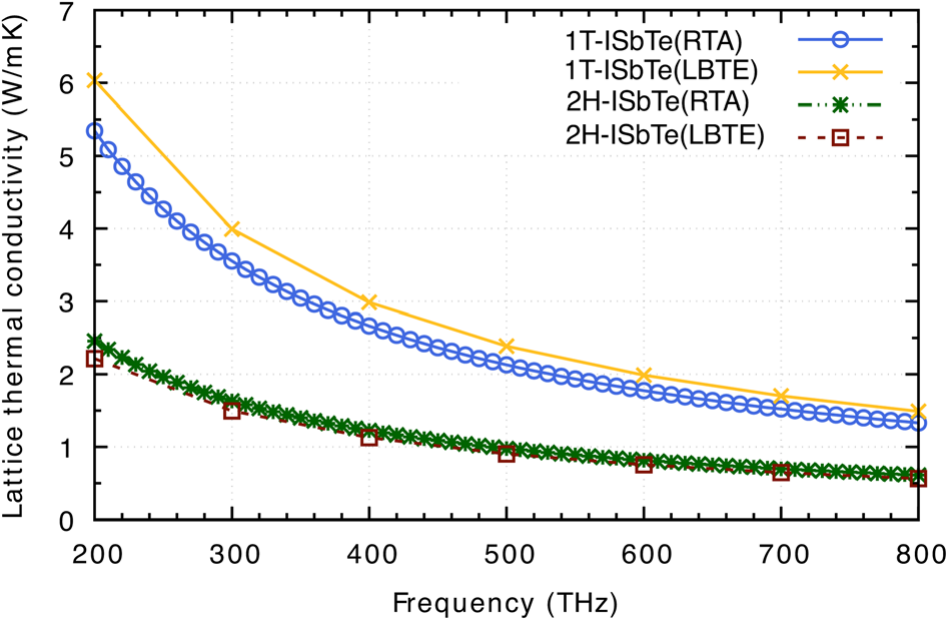
Calculated lattice thermal conductivity *κ*_L_ of 1T-ISbTe and 2H-ISbTe using RTA and LBTE. The RTA results, which need less computational time and memory, are computed at more temperature points, whereas the LBTE results are computed with a temperature step of 100 K. The present results are already normalized to the vacuum size and the thickness of the monolayer (see text).

Having all the ingredients to obtain the *κ*_L_ from eqn (4), we can now discuss the differences in the *κ*_L_ between the two structures of interest. Fig. 8 shows the calculated *κ*_L_ of 1T and 2H-ISbTe using the relaxation time approximation (RTA) and direct-solution of phonon Boltzmann equation (LBTE). For 1T-ISbTe, the LBTE is slightly higher than that of RTA and exhibits a *κ*_L_ of 3.55 (4.00) W mK^−1^ using RTA (LBTE). The 2H-ISbTe result is less sensitive to *κ*_L_ calculation methods, exhibiting values of 1.63 and 1.5 W mK^−1^ for RTA and LBTE, respectively. The different values of *κ*_L_ in the 2 phases are large at low temperatures and gradually reduce when the temperature increases. Both cases have low isotropic lattice thermal conductivity as compared to other 2D materials such as TMDs^54,55^ and Janus materials,^56,57^ and stand among the promising thermoelectric materials in low dimensions.^58,59^

**Fig. 9:**
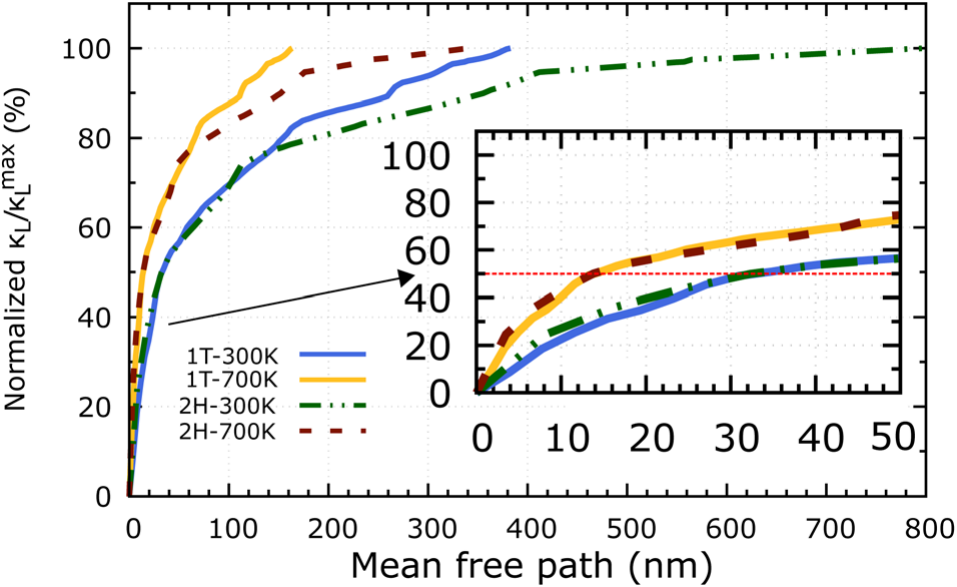
Cumulative *κ*_L_ as a function of the phonon mean free path of 1T and 2H-ISbTe at 300 K and 700 K. The inset figure displays identical data with MFPs smaller than 50 nm. The dashed red line denotes the 50% as a guide for eye.

Apart from the phonon spectrum, analysing the distributions of phonon mean free paths (MFPs) can provide us a more complete view of phonon transport. We can learn about the size-dependent thermal transport and the origins of the lattice's low thermal conductivity from these. Fig. 9 shows the cumulative *κ*_L_ as a function of phonon MFPs. When only phonons with an MFP below a specific threshold are used in the calculations, this number represents the hypothetical lattice thermal conductivity. In particular, we focus on the percentage amount of total *κ*_L_ accumulation (normalised to the *κ*^max^_L_) that can be attributed to phonons with MFPs below the threshold. The 2H-ISbTe and 1T-ISbTe phonon mean free paths are 800 nm and 383 nm, respectively, which is shorter than other monolayer TMDs.^55^ Significantly, the 50% *κ*_L_ accumulation MFP values at room temperature are 14 nm and 32 nm for 1T and 2H-ISbTe respectively, as shown in the inset of Fig. 9, which allows room to further reduce *κ*_L_ by nanostructuring. To significantly reduce *κ*_L_ below its fundamental values, nanostructures with typical diameters below 14 nm and 32 nm for 1T and 2H-ISbTe, respectively, would be required. We further demonstrate the temperature effect of MFPs by showing that at both 300 K and 700 K, the contribution of phonons with MFPs that allow a 50% reduction in *κ* − *L* is found to be about the same. This result suggests that the same amount of effort required for phonon transport at low temperatures may be used when nanostructuring materials to operate at higher temperatures.

**Fig. 10:**
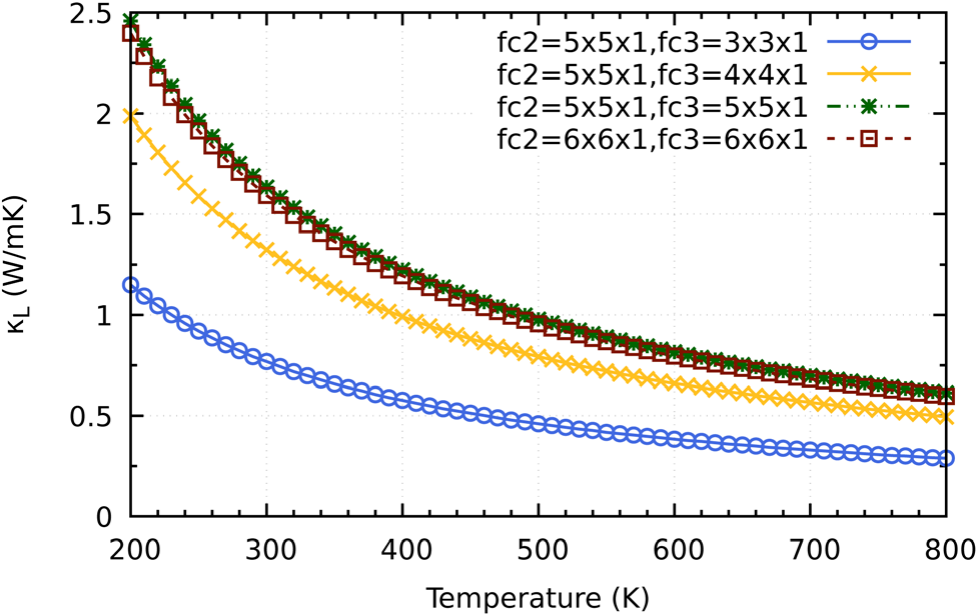
Supercell size influence on the convergence of *κ*_L_ calculations for 2H-ISbTe. Fc2 is the supercell size for second order force constant and fc3 is for the third order force constant.

Before coming to the conclusion of our work, we would want to explore the significance of the supercell size impact on the convergence *κ*_L_ calculation. As shown in Fig. 10 a small supercell size such as 3 × 3 × 1 or 4 × 4 × 1 for calculating the third order force constants would clearly underestimate the converged values of 2H-ISbTe. At 300 K, the values are 0.77 and 1.32 W mK^−1^ for 3 × 3 × 1 and 4 × 4 × 1, respectively. The results we have presented use a supercall size of 5 × 5 × 1 which converges nicely with the larger cell 6 × 6 × 1 to avoid slipping into the misleading conclusion of ‘ultralow’ values that the smaller supercell size may demonstrate.

## 4 Conclusion

Using first-principles calculations and the Boltzmann transport equation accelerated by machine learning interatomic potential, we investigated the phonon transport characteristics of Janus ISbTe siblings. 2H-ISbTe is found to have a substantially lower *κ*_L_ than 1T-ISbTe. Although the two siblings share a comparable covalent boding and low elastic constant, their phonon group velocity and lifetime distinguish them significantly. Sb atoms are the dominant heat carriers in 2H-ISbTe, whereas I atoms dominate the phonon transport in 1T-ISbTe, as shown by an exhaustive analysis of their phononic properties. In addition, 1T-ISbTe has a peculiar property where its optical modes contribute significantly to *κ*_L_. Nanostructuring is found to be simpler in 2H-ISbTe than in 1T when the sample size is 32 nm in 2H-ISbTe *versus* 14 nm in 1T. In addition to shedding light on the behavior of these specific Janus monolayers, our results encourage both the experimental and theoretical development of two-dimensional materials as high-performance thermoelectric materials.

## Author contributions

Viet-Ha Chu: writing – original draft, methodology. Tien-Ha Le and Truong-Tho Pham: formal analysis, investigation, Duc-Long Nguyen: conceptualization, writing – review & editing, visualization, supervision.

## Conflicts of interest

The authors declare that they have no conflict of interest.

## Supplementary Material
